# Analysis and Optimization of Thermodiffusion of an FBG Sensor in the Gas Nitriding Process

**DOI:** 10.3390/mi7120227

**Published:** 2016-12-12

**Authors:** Tso-Sheng Hsieh, Yi-Chian Chen, Chia-Chin Chiang

**Affiliations:** Department of Mechanical Engineering, National Kaohsiung University of Applied Sciences, Kaohsiung 807, Taiwan; srcx2s904@gmail.com (T.-S.H.); al11062000@gmail.com (Y.-C.C.)

**Keywords:** temperature sensitivity, gas nitriding process, fiber Bragg grating, thermo-optic coefficient

## Abstract

In this paper, we report the numerical calculations for a thermo-optical model and the temperature sensitivity of a fiber Bragg grating (FBG) sensor. The thermally-induced behaviors of a FBG sensor in the gas nitriding process were analyzed for temperatures ranging from 100–650 °C. The FBG consisted of properly chosen photosensitive fiber materials with an optimized thermo-optic coefficient. The experimental and optimized thermo-optic coefficient results were consistent in terms of temperature sensitivity. In these experiments, the temperature sensitivity of the FBG was found to be 11.9 pm/°C.

## 1. Introduction

With the development of distributed fiber-based temperature and strain sensors, the integration of optical fibers (OF) has been further encouraged in harsh environments such as nuclear power plants and fusion and high energy physics facilities [1]. The applications of fiber Bragg grating (FBG) temperature sensors are increasing daily due to the advantages these sensors offer in terms of electromagnetic interference and polarization, in addition to their being corrosion resistant, light-weight, low-cost, and long-lasting—among other positive attributes [2,3,4,5]. Bare FBG sensors cannot be used in the measurement of temperatures, because they are made of glass or silica—the thermal expansion coefficients of which are very low [6]. At telecommunication wavelengths near room temperature, the wavelengths of such FBG sensors shift with changes in temperature at a rate of approximately 9.4 pm/°C [7]. One problem with normal FBGs is thus their temperature stability [8], with typical FBGs being stable below 300–400 °C [9]. Butov et al. [10] reported on an FBG composed of nitrogen-doped silica fibers with high energy density per pulse—a type of fiber that was used to obtain a thermo-resistant character for high temperature applications. The limitation of this FBG is that high energy density per pulse is required in the grating writing process. Therefore, other FBG fabrication methods cannot be used to produce this thermo-resistant FBG [10]. It should also be noted that Type I gratings made with nitrogen-doped fibers were previously found to decay fully at temperatures of 600–700 °C.

The mathematical modeling of fiber grating is mostly accomplished using the coupled mode theory of wave propagation and the transfer matrix method [11,12]. Mukesh et al. [13] conducted a simulation study of coated FBG used as a temperature sensor using the GratingMOD of RSoft Photonics computer-aided design (CAD) Suites (version 2013.12, Synopsys, Mountain View, CA, USA), where α was the thermal expansion coefficient of silica and was equal to 0.55 × 10^−6^, while ξ was the thermo-optic coefficient of the fiber material and was equal to 8.3 × 10^−6^. Meanwhile, a pure quartz fiber has a different ξ of 6.67 × 10^−6^ [14]. On a related note, because of the differences among different fibers—including differences in the ways that fiber gratings are etched and annealed [15,16]—the performances of different FBG sensors in terms of temperature sensitivity can be very different. In this study, we determined the high temperature decay mechanisms of FBGs surrounded by nitrogen gas in the context of the gas nitriding process, and measured the Bragg wavelength shift and temperature sensitivity of these FBGs. Finally, the measurement results were than compared and verified against results obtained using the finite element method (FEM).

## 2. Principle of Fiber Bragg Grating (FBG) Sensors

The type of fiber investigated in this study had the following characteristics: an inner core with a diameter of 9.6 μm and an outer cladding of glass (SiO_2_) with a diameter of 124.9 μm encased in photosensitive (PS) 1250/1500 (Fibercore) fiber. The FBG writing method used to produce the FBG is based on phase mask (type I) exposition to a KrF excimer laser (Coherent Xantos XS, 248 nm wavelength, 12 mJ/cm^2^, Coherent, Palo Alto, CA, USA), and the grating peak reflectivity was about 97%.

Thermally-induced changes in the index and the grating period of the FBG induce a shift in the Bragg wavelength of the grating. This characteristic Bragg wavelength shift is determined by the effective refractive index and the grating period, and can be defined as

(1) $$\lambda_{B1}=2n_{\mathrm{eff}}\Lambda$$

where *n*_eff_ is the effective refractive index, Λ is the grating pitch, and the subscript “1” is the initial state before the temperature change.

Furthermore, individual effects can be superimposed to obtain the Bragg wavelength shift.

(2) $$\frac{{\Delta \lambda }_{B}}{\lambda_{B1}}=\frac{\Delta n_{\mathrm{eff}}}{n_{\mathrm{eff}}}+\frac{\Delta \Lambda }{\Lambda }$$

where Δ*n*_eff_/*n*_eff_ is the change in the effective refractive index resulting from a change in temperature and ΔΛ/Λ is the contribution of thermal expansion due to the gas nitriding process.

The index of refraction for the core and cladding of the fiber used in this study is among the smallest of those for all glass optical fibers. The derivative of the effective index of FBG with respect to temperature can be taken to be equal to the *dn*/*dT* of the fiber core material, and the change in the effective index of refraction is given by

(3) $$\Delta n_{\mathrm{eff}}=\frac{dn}{dT}\Delta T$$

where *dn*/*dT* is the thermo-optical coefficient and Δ*T* is the change in temperature.

The relative change of the grating period due to thermal expansion is given by

(4) $$\frac{\Delta \Lambda }{\Lambda }=\alpha \Delta T$$

where α is the thermal expansion coefficient.

The thermo-optical model was analyzed in terms of the effects of the temperature field on the optical characteristics. The Bragg wavelength shift λ_B2_ can be written as

(5) $$\lambda_{B2}=\lambda_{B1}+\lambda_{B}=2\left(n_{\mathrm{eff}1}+n_{\mathrm{eff}}\right)\left(\Lambda_{1}+\Delta \Lambda \right)$$

(6) $$\lambda_{B2}=2\left(n_{\mathrm{eff}1}+\frac{dn}{dT}\right)\left(\Lambda_{1}+\Lambda_{1}\alpha \Delta T\right)$$

The analytical formula for the normalized Bragg wavelength shift can be expressed as

(7) $${\Delta \lambda }_{B}=\lambda_{B1}\left(\frac{1}{n_{\mathrm{eff}}}\frac{\Delta n_{\mathrm{eff}}}{\Delta T}+\frac{1}{\Lambda }\frac{\Delta \Lambda }{\Delta T}\right)\Delta T=\lambda_{B1}\left(\alpha +\xi \right)\Delta T$$

By optimizing the core material, the value of α can be adjusted to approximately 0.55 × 10^−6^, and the value of can be adjusted to approximately 7.18 × 10^−6^. The thermo-optic coefficient and thermal expansion coefficient in the temperature coefficient can be written as β = α + ξ, where β is the constant material parameter.

## 3. Experimental

### 3.1. Measurement of FBG Sensor Performance

**Figure 1:**
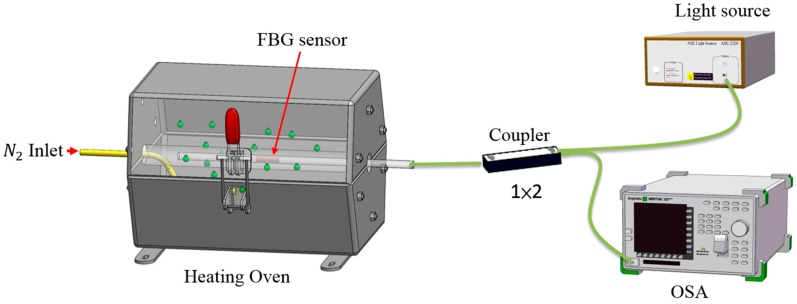
The experimental set-up of the temperature sensing charged nitriding process. FBG: fiber Bragg grating, OSA: optical spectrum analyzer.

The FBG sensors were placed into a heating oven filled with nitriding in order to study and compare their responses to changes in temperature. The experimental set-up is illustrated in Figure 1. A broadband light ray was injected using a super luminescent diode (SLD) broadband light source. A jumper wire was connected to one end of the 1 × 2 optical coupler, while the other end could be connected to super-fluorescent fiber source with a spectrum analysis instrument. The reflected spectra of the FBG sensors were scanned and stored via an optical spectrum analyzer (OSA). The bare FBG sensor (1556.04 nm and 23 °C) and bare FBG sensor surrounded by nitrogen gas (1556.40 nm and 23 °C) were placed into the heating oven one at a time, which was certainly capable of influencing the data quality of the test experiments in terms of the signals emitted. The temperature variation range was from 100 to 650 °C. The data were recorded at 50 °C intervals. Every temperature point was maintained for 10 min in order to record the reflective spectrum of the relationship between the wavelength and the temperature. The average value of 10 recordings for a given temperature was used as the test result for that temperature.

### 3.2. Simulation of FBG Sensor

The R-soft software was then employed to analyze the temperature sensitivity of the FBGs at various surrounding temperatures. For experimentation, the R-soft software was initially employed to confirm the temperature sensitivity values measured by the FBG for the various surrounding temperatures by comparing the FBG change results with the Bragg wavelength shifts at the various temperatures. The results were then used to analyze the thermal expansion coefficient and the thermo-optic coefficient of the FBG temperature sensor.

Default waveguide settings were set in order to produce a standard single mode fiber with a diameter of 9.6 μm, a cladding index of 1.466, and a core index of 1.474. This corresponded to the following CAD parameters (as shown in Table 1): index difference = 0.008, background index = 1.466, and component width = component height = 9.6 µm. Two typical free space wavelengths of 1556.70 and 1557.00 nm were used at 100 °C, while the simulation test temperature ranged from 100 to 650 °C. The grating perturbation—defined as the way the waveguide parameters vary along the propagation direction—was 0.0003. The thermo-optic coefficient ξ and thermal expansion coefficient α of the fiber were defined as Dn/*dt* and DΛ/*dt* in the RSoft CAD layout parameters.

Lastly, in order to indicate that a gratingmod simulation was to be performed, the simulation tool was set to gratingmod. The OK button was then pressed to start the design process.

## 4. Results and Discussion

For the high temperature test of the bare FBG sensor surrounded by nitrogen gas, the temperature sensitivity and responsivity of the bare FBG sensor in the oven was measured as light was injected into the fiber core by the broadband laser. The FBG sensor measured the temperature sensitivity, which is launched into the fiber core by the broadband laser. The thermal expansion coefficient of the bare FBG sensor and the refractive index in the grating changed as the temperature was increased. The changes in the temperature could be observed via the reflection spectra of the FBG, which are themselves the result of perturbations in the gratings resulting in shifts in the Bragg wavelength.

### 4.1. Comparison of the Bare FBG Sensors with and without Nitriding

**Figure 2:**
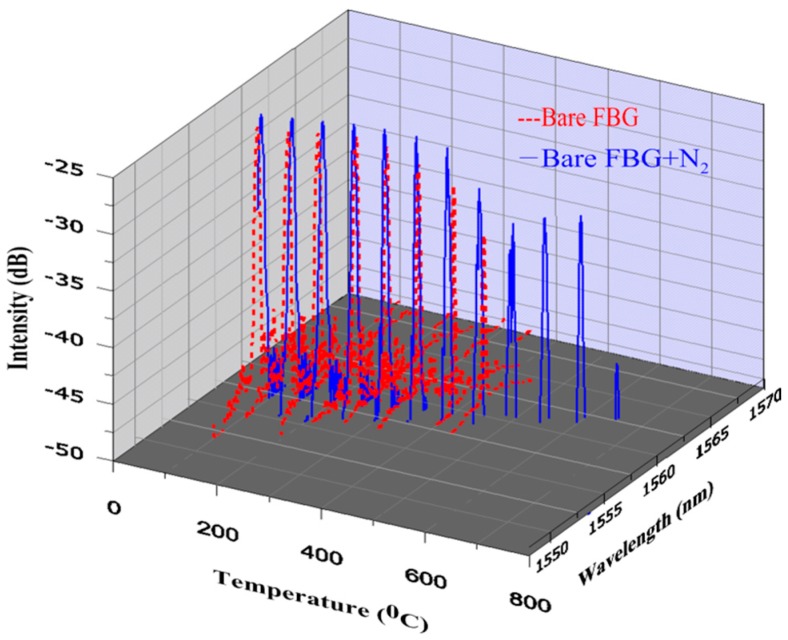
The online monitoring diagram of the highly reflective bare FBG surrounded by gas produced by the gas nitriding process and the bare FBG not surrounded by gas produced by the gas nitriding process.

**Figure 3:**
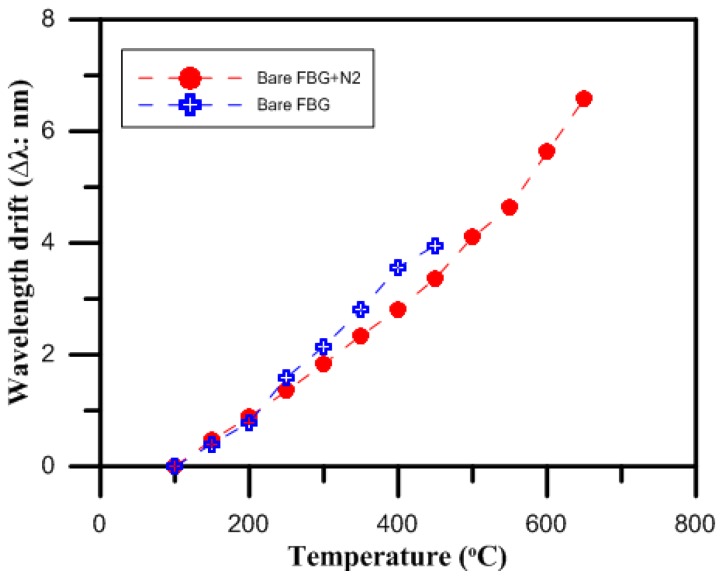
Temperature characteristics of the bare FBG surrounded by gas produced by the gas nitriding process and the bare FBG not surrounded by gas produced by the gas nitriding process.

Figure 2 shows the intensity spectra results for the FBG both with and without nitriding as the temperature was changed and the Bragg wavelength shifted. The shifts in the Bragg wavelength, Δλ*B*, for both the charged and uncharged nitriding bare FBG at the different temperatures were then compared. A comparison of the temperature sensitivities of the bare FBG surrounded by gas produced by the gas nitriding process and the bare FBG not surrounded by gas produced by the gas nitriding process is shown in Figure 3. The results showed that the intensity spectrum of the bare FBG without nitrogen at 100 °C was measured to be 1556.70 nm, while that of the bare FBG surrounded by nitrogen gas from the gas nitriding process at the same temperature was measured to be 1557.00 nm. Moreover, the sensor parameter of the bare FBG not surrounded by gas produced by the gas nitriding process effectively disappeared for the temperature range from 500 to 650 °C, indicating that the fiber had failed. The results also showed that the shift in the Bragg wavelength for the bare FBG not surrounded by gas produced by the gas nitriding process was 3.96 nm over the temperature range from 100 to 450 °C, while that for the bare FBG surrounded by gas produced by the gas nitriding process for the same range was 3.38 nm. In addition, the shift in the Bragg wavelength for the bare FBG surrounded by gas produced by the gas nitriding process was 3.22 nm over the temperature range from 450 to 650 °C.

**Figure 4:**
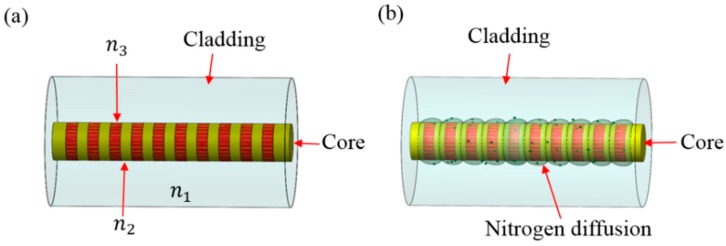
The thermodiffusion from the irradiated pixels of the grating during high-temperature treatment (**a**) FBG sensor and (**b**) FBG sensor during the gas nitriding process.

**Figure 5:**
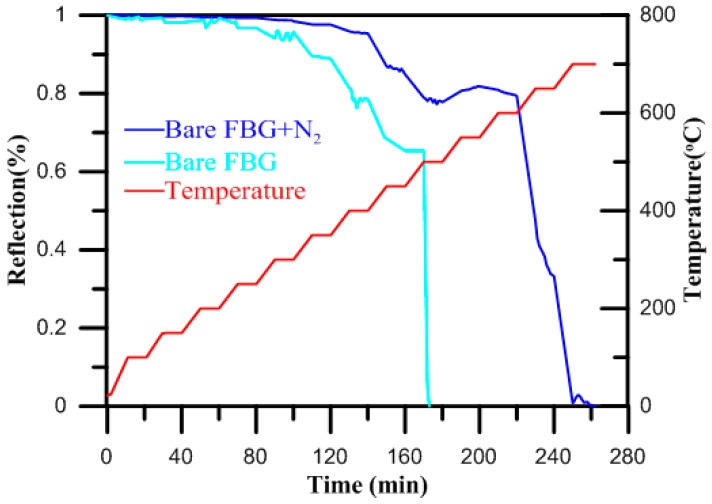
Reflectivity evolutions during thermal annealing of the bare FBG subjected to the gas nitriding process and the bare FBG subjected to the gas nitriding process.

During the annealing process, one of the weakest bonds among the three bonds of the drawing-induced defect (DID) will be broken by annealing energy. Therefore, the thermal energy-induced structural change from the DID into the germanium-oxygen-deficient center (GODC) is the principal cause of the thermal decay of FBG refractivity [17]. The effect of the nitriding process on thermodiffusion is the reason for the increased reflectivity in high temperature gratings annealing. To all appearances, this can be explained by a nearly twofold reduction in the distance between grating pixels. In these conditions, the diffusion of gas produced by the gas nitriding process immediately leads to grating degradation, resulting in a slight increase in the fiber core diameter [18]. Figure 4 shows the gas nitriding diffusion from the irradiated pixels of the grating during high-temperature treatment. The structural diagram of the FBG is shown in Figure 4. The refractive indices of the cladding, core and surrounding (gas nitriding process) are *n*1, *n*2, *n*3, respectively. The variation in the fill gas and temperature refractive index response to the surrounding will cause a shift of the spectrum of the FBG. In Figure 4a, a bare FBG sensor made of boron (B/Ge) co-doped photosensitive fiber is illustrated. The absorption of gas produced by the gas nitriding process by the core/cladding interface, and the resulting formation of a compound are shown in Figure 4b. When the FBG sensor layer comes in contact with gas produced by the gas nitriding process, it causes swelling, stress, and strain on the core. The wavelength then shifts due to the resulting stretch and strain in the FBG, and this shift can be detected using an optical spectrum analyzer. Optical heating of the FBG during the gas nitriding process is important to protect its sensitivity. Reflectivity evolutions during thermal annealing of the bare FBG subjected to the gas nitriding process and the bare FBG not subjected to the gas nitriding process are shown in Figure 5. It can be observed that the reflectivity of the bare FBG subjected to the gas nitriding process was higher than that of the bare FBG not subjected to the gas nitriding process at the same temperature. The sensor parameter of the bare FBG not subjected to the gas nitriding process effectively disappeared for the temperature range from 500 to 650 °C, but the bare FBG subjected to the gas nitriding process continued working in the same temperature range.

**Figure 6:**
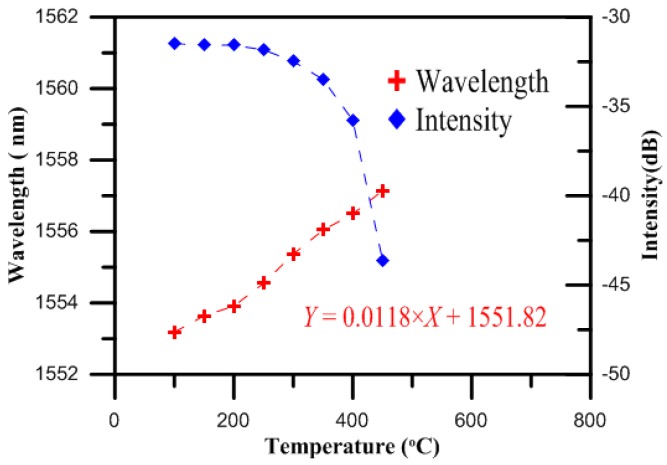
Temperature characteristics of the FBG pre-exposed to nitrogen.

To clarify whether or not the effect that nitrogen exposure makes the FBG survive elevated temperatures is permanent, we pre-exposed the FBG to nitrogen for one hour, and then started to heat up the fiber. The wavelength of the bare FBG was measured to be 1553.40 nm when the temperature of 100 °C was reached, after which the inlet for the nitrogen was closed off. The wavelength of the FBG pre-exposed to nitrogen disappeared at 475 °C, as shown in Figure 6. The nitrogen exposure caused the FBG to continue functioning at the elevated temperatures, but the effect was not permanent.

### 4.2. Analysis Using the Finite Element Method (FEM)

**Figure 7:**
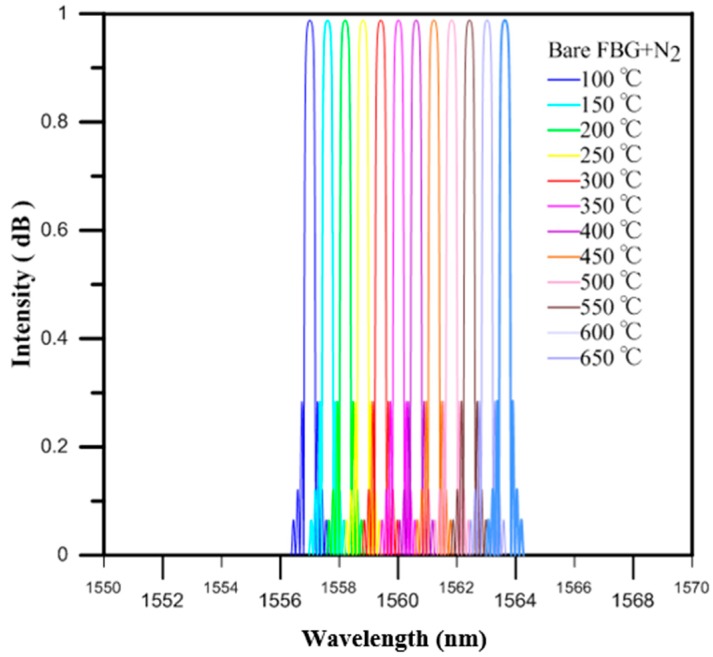
The online monitoring diagram of the highly reflective bare FBG in the RSoft simulation.

The design of the optical fiber temperature sensor was proven to be reasonable by the experimental results. Then, finite element analysis software (RSoft) was used for the temperature test simulation, with the temperature ranging from 100 to 650 °C in intervals of 50 °C per step. The monitor step size and slice step size were equal to 0.25 per period. The shifts in the Bragg wavelength of the bare FBG surrounded with charged nitrogen produced by the gas nitriding process for the temperatures from 100 to 650 °C are shown in Figure 7. The thermo-optic coefficient (ξ = 6.67 × 10^−6^) of the fiber was previously reported in another study [14]. From the results of the RSoft simulation, and by optimizing the thermo-optic coefficient of the core material, we obtained a value of approximately 7.18 × 10^−6^, which was closer to that of the experimental results.

**Figure 8:**
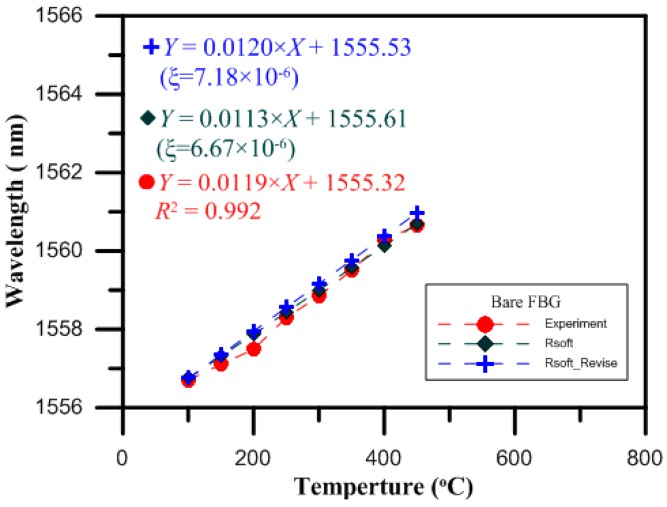
Temperature characteristics of the bare FBG sensors in the RSoft simulation and in the experiment without nitriding.

The values measured by the bare FBG temperature sensor for loadings of 0 to 450 °C were verified by the uncharged nitriding experiment measurements. When the temperature was 450 °C, the drift of the wavelength strength variation was 3.96 nm, and the sensitivity of this temperature sensor was 11.9 pm/°C, while the wavelength variation of the RSoft simulation analysis was also proportional to the temperature loading, as shown in Figure 8.

**Figure 9:**
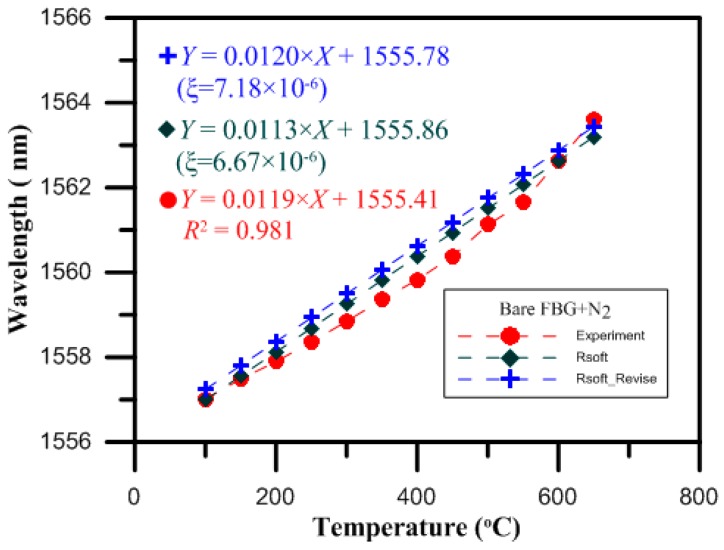
Temperature characteristics of the bare FBG sensors in the RSoft simulation and in the experiment with nitriding.

The values measured by the bare FBG temperature sensor for loadings of 0 to 650 °C were verified by the charged nitriding experiment measurements. When the temperature was 650 °C, the drift of the wavelength strength variation was 6.60 nm, and the sensitivity of this temperature sensor was 11.9 pm/°C, while the wavelength variation of the RSoft simulation analysis was also proportional to the temperature loading, as shown in Figure 9.

It should be noted that the exponential points of the dependence of the Bragg wavelength shift on temperature measured in germanosilicate fiber gratings do not strictly obey a linear law either, and this is probably associated with the effects of gas nitriding thermodiffusion at 350 to 600 °C.

## 5. Conclusions

In this study, we have presented a temperature sensitivity results for bare FBG subjected to the gas nitriding process by calculating the relevant thermo-optic parameters. The thermo-optic coefficient was 7.18 × 10^−6^, and the temperature sensitivity was 11.9 pm/°C. Comparison of the experimental results and simulated results for the bare FBG subjected to the gas nitriding process were obtained using thermodiffusion sensing capabilities for high temperature. The temperature sensitivity of the bare FBG subjected to the gas nitriding process was better than that of the bare FBG not subjected to the gas nitriding process at temperatures above 450 °C. In addition, the bare FBG subjected to the gas nitriding process continued working at temperatures of up to 650 °C. Experimental characterizations of the temperature sensitivity and the optimized thermo-optic coefficient were consistent in terms of temperature sensitivity.

## Figures and Tables

**Table 1:** The simulation parameters of the fiber Bragg grating (FBG). GratingMOD: the module name.

Simulation Tool	GratingMOD	Simulation Tool	GratingMOD
Grating type	Volume index	Width	9.6 µm
Structure type	Fiber	ModDelta	0.0003
Index profile	Step index	Delta	0.008
Length	5000 µm	Dn/dt (ξ)	7.18 × 10^−6^
Height	9.6 µm	DΛ/dt (α)	0.55 × 10^−6^
